# pH-responsive chitosan–silica nanoparticles for salicylic acid delivery to alleviate continuous cropping stress in ginseng cultivation

**DOI:** 10.3389/fmicb.2025.1671037

**Published:** 2025-12-11

**Authors:** Jiajun Wang, Ling Li, Sizhang Liu, Baowei Feng, Zhefeng Xu, Huan Wang, Tao Zhang, Xue Chen, Qiong Li, Changbao Chen

**Affiliations:** Jilin Ginseng Academy, Changchun University of Chinese Medicine, Changchun, China

**Keywords:** ginseng, continuous cropping obstacles, chitosan-modified mesoporous silica nanoparticles, salicylic acid, microbial community, soil amendment

## Abstract

Continuous ginseng cultivation leads to soil degradation, which adversely affects seed germination and seedling growth. To address this issue, we developed chitosan-modified mesoporous silica nanoparticles loaded with salicylic acid (SA), hereafter referred to as CS@MSN-SA, as an innovative soil amendment. With a diameter of approximately 200 nm, these hollow mesoporous nanocarriers mediated the pH-responsive controlled release of SA under neutral and acidic conditions. Ginseng seeds were sown under four soil conditions: (1) new soil, (2) continuous cropping soil, (3) new soil treated with CS@MSN-SA, and (4) continuous cropping soil treated with CS@MSN-SA. A comprehensive analysis was conducted on the rhizosphere soil, including microbial community structure and diversity, carbon source metabolic potential, nutrient content, and enzymatic activities. The results showed that CS@MSN-SA treatment significantly enhanced the growth parameters and antioxidant activity of ginseng compared to the untreated continuous cropping soil. CS@MSN-SA improved soil nutrient levels (N, P, K, available forms, and organic matter), increased pH, and boosted enzymatic activity. It also promoted beneficial shifts in microbial community structure and richness while increasing the microbial utilization of amines, carbohydrates, and carboxylic acids. Statistical analysis revealed a significant correlation between microbial community abundance and soil property parameters. Overall, CS@MSN-SA nanoparticles effectively mitigated the negative effects of continuous cropping by improving soil fertility and microbial balance, thereby promoting seed germination and healthy seedling development during ginseng cultivation.

## 1 Introduction

Ginseng (*Panax ginseng* C. A. Meyer), commonly known as ginseng, is a valuable medicinal plant that is widely cultivated for its diverse pharmacological effects, including antioxidant, anti-tumor, and immune-enhancing properties (Kim et al., 2019; Lee et al., 2025). With a steadily increasing demand in the global market (Ratan et al., 2020), sustainable cultivation of ginseng has become increasingly important. However, continuous cropping obstacles (CCO) have become a major constraint in ginseng production, primarily driven by allelochemical accumulation, microbial dysbiosis, and soil property deterioration (Shen et al., 2024). Microbial dysbiosis has been identified as the direct cause of CCO (Wang et al., 2024). Microbial dysbiosis—marked by an increase in pathogens and a decline in beneficial microbes—disrupts nutrient cycling, diminishes biological activity, and promotes autotoxin accumulation, collectively leading to long-term soil degradation. These interactions form a self-perpetuating “allelochemicals–pathogens–soil environment” feedback loop that severely reduces ginseng yield and quality (Miao et al., 2023; Bischoff Nunes and Goodwin, 2022). This process develops progressively; studies have shown that after just 2 years of continuous cultivation, significant changes in microbial diversity and abundance occur in the soil (Xu et al., 2025). Therefore, microbial remediation is essential during both the initial planting and replanting phases of ginseng cultivation.

Salicylic acid (SA) is a natural endogenous plant hormone with no known ecotoxicity. At low concentrations, it can promote plant growth as a growth hormone, whereas at high concentrations, it inhibits pathogenic bacteria by affecting membrane proteins (Bandara et al., 2016) and promotes plant resistance. Additionally, SA can regulate immune signal transduction to select specific bacterial taxa for colonization around plant roots, thereby exerting a significant impact on shaping the rhizosphere microbial community (Lebeis et al., 2015). SA demonstrates dual functionality by modulating soil microbial communities and inducing systemic resistance in plants, highlighting its potential as a multifunctional soil amendment. However, its application is limited by the fact that it is easily degraded by soil microorganisms. Most previous studies have adopted SA spraying, which can activate the defense response of ginseng and promote its growth (Thilakarathne et al., 2025). However, this method is limited by its short effective duration and inability to precisely control its concentration, resulting in restricted efficacy against CCO during replanting. In recent years, nanotechnology has offered a promising solution to this issue. Mesoporous silica nanoparticles (MSN), with their high specific surface area, excellent biocompatibility, and loading capacity, can serve as ideal carriers for active substances. MSN can regulate soil microecology while supplementing nutrient elements and enhancing plant resistance (Bian et al., 2024). Facilitating the controlled release of SA in soil via MSN offers a promising approach for employing SA as a soil amendment. However, CCOs are often accompanied by soil acidification, even when the pH drops to around 5.0 (Wang et al., 2025; Matsumoto et al., 2022). However, MSNs lack an intelligent release design tailored to acidic environments.

In this study, a pH-responsive nanomaterial, chitosan-modified MSN loaded with SA (CS@MSN-SA), was designed. CS@MSN-SA was constructed by wrapping a chitosan (CS) coating around the core of SA-loaded MSN. Under neutral conditions, positively charged CS binds to negatively charged MSNs through electrostatic interactions, thereby encapsulating SA. Under acidic conditions, H^+^ disrupts electrostatic interactions, triggering swelling of the material and rapid release of SA. We systematically characterized the physicochemical properties of CS@MSN-SA and its pH-dependent release behavior. Additionally, pot experiments were conducted using ginseng soil that had been continuously cropped for 5 years and unused new soil. The effects of this material on ginseng germination and growth, rhizosphere microbial community, and soil properties were comprehensively evaluated to assess its potential for improving soil health and alleviating CCO. The functional mechanism of CS@MSN-SA was preliminarily explored and elucidated, hoping to provide new methods and ideas for resolving replanting issues.

## 2 Materials and methods

### 2.1 Preparation of nanoparticles

MSN was synthesized according to a conventional optimized sol–gel method (Zhang et al., 2024). To remove the residual CTAB template, the synthesized powders were calcined at 550 °C for 6 h in a muffle furnace and then cooled to room temperature under an anhydrous atmosphere to obtain pure MSN. Subsequently, MSN (0.5 g) was dispersed in a 20% SA ethanol solution (50 ml) and stirred at 400 rpm for 24 h. The resulting particles were collected by centrifugation (8,000 rpm, 10 min), washed thoroughly with deionized water to remove free SA, and dried under vacuum to obtain SA-loaded MSN (MSN-SA). For the CS coating, MSN-SA (0.5 g) was dispersed in a 2% CS aqueous solution (50 mL, acidified with 200 μL of 38% HCl) under stirring at 200 rpm for 24 h. The final product, CS@MSN-SA, was harvested by centrifugation (8,000 rpm, 10 min), purified via three washes with deionized water, and lyophilized.

#### 2.1.1 The physicochemical properties of nanoparticles

A scanning electron microscope (SEM, Quattro S, Thermo Fisher Scientific, US) was used to observe the surface morphology of the nanoparticles and assess their dispersion state and nanoparticle size. A transmission electron microscope (TEM, Tecnai F20, FEI Company, US) was used to analyze the microstructure of the MSN, internal pore distribution, and pore size. Brunauer–Emmett–Teller (BET, ASAP 2460, Micromeritics Instrument Corporation, US) testing was used to accurately calculate the pore size distribution and specific surface area of the MSN. A dynamic light scattering analyzer (DLS, Nano ZS90, Malvern Panalytical, UK) was used to accurately determine the particle size distribution of the nanoparticles. X-ray diffraction (XRD, XRD-6100, Shimadzu Corporation, Japan) was used to analyze the crystal structures of MSNs and CS@MSN-SA. Fourier-transform infrared spectroscopy (FT-IR, Nicolet iS5, Thermo Fisher Scientific, US) was used to identify the organic functional groups modified on the surface of CS@MSN-SA.

#### 2.1.2 SA loading and release capacity of CS@MSN-SA

The SA loading and release capacity of CS@MSN-SA were evaluated using ultraviolet–visible (UV–Vis) spectroscopy. A calibration curve was constructed using a series of SA standard solutions at concentrations of 0, 50, 100, 150, 200, and 250 μg/mL. The optical density of each solution at 298 nm (OD_298_) was measured using a UV–Vis spectrophotometer (Synergy H1, Agilent BioTek, US). The standard curve was plotted with SA concentrations on the x-axis and OD_298_ values on the y-axis. The data exhibited a strong linear relationship, and the regression equation was calculated as y = 0.0084x + 0.6161 (*R*^2^ = 0.9978, *n* = 3, over the range of 0–250 μg/mL), where y is the measured absorbance and x is SA concentration (μg/mL).

Salicylic acid loading capacity of CS@MSN-SA. CS@MSN-SA (20 mg) was dispersed in an aqueous solution (50 mL) and subjected to ultrasonic extraction of SA at 25 °C for 2 h. The OD_298_ value of the extraction solution was measured, and the SA loading capacity was calculated using the standard curve equation.

Salicylic acid released the capacity of CS@MSN-SA. CS@MSN-SA (60 mg) was added into a dialysis bag (MWCO 10000), which was separately immersed in an aqueous solution (200 mL) at pH = 7 and 5, maintained at 25.0 ± 0.5 °C for 24 h under constant agitation. The OD_298_ value of the solution was monitored, and the SA-released content was calculated.

Control Experiment: Dissolution time of pure SA. SA (50 mg) was dissolved in deionized water (200 mL), and the OD_298_ of the SA solution was monitored until the OD_298_ value began to plateau. The time at which the plateau was first reached was recorded as the complete dissolution time.

#### 2.1.3 CS encapsulation efficiency of CS@MSN-SA

Standard curve establishment. CS content was measured using the phenol-sulfuric acid method. CS solutions at various concentrations of 0, 50, 100, 150, 200, and 250 μg/mL were prepared. After the addition of phenol and sulfuric acid, the OD_490_ value of the reaction solution was determined by UV–visible spectroscopy after being cooled to room temperature. The standard curve was plotted with the CS solution content as the x-axis and the corresponding OD_490_ value as the y-axis. The standard curve equation of CS content was expressed as *y* = 0.0139x + 0.0502 (*R*^2^ = 0.996, *n* = 3, over the range of 0–250 μg/mL).

CS encapsulation efficiency of CS@MSN-SA. CS@MSN-SA (5 mg) was dispersed in 50 mL of aqueous solution and extracted at 25 °C for 2 h by the ultrasonic method. The CS encapsulation amount was calculated by applying the measured OD_490_ value to the standard curve equation of the CS content.

### 2.2 Agronomic traits and antioxidant bioactivity characterization of ginseng seedlings

#### 2.2.1 Pot experiment

Continuous ginseng cultivation soil for 5 years was collected from Sanhe Village, Dashitou Town, Dunhua City, Jilin Province, China. New soil samples were collected from adjacent farmlands with no history of ginseng cultivation. The pot experiment was conducted in May 2024 in an artificial climate chamber at Changchun University of Chinese Medicine under controlled conditions of a 14-h/10-h light/dark cycle, 25 ± 2 °C, and 60 ± 5% relative humidity. Each plastic pot (9 cm × 9 cm × 8 cm) was filled with 300 g of soil and sown with 25 ginseng seeds. The seeds, procured from the FuSong market in Tonghua City, Jilin Province, China, were divided into four treatment groups: (1) continuous cultivation ginseng soil (CCGS), (2) CCGS amended with 0.25 g of CS@MSN-SA nanoparticles (CCGS-nps), (3) new soil (NS), and (4) NS amended with 0.25 g of CS@MSN-SA nanoparticles (NS-nps). The nanoparticles were thoroughly mixed with soil prior to potting. Each treatment group was randomly assigned to one of five pots (n = 5). Ginseng seedlings were harvested on the 40th day. Five randomly selected seedlings from each treatment group were collected for the analysis of morphological indices and antioxidant enzyme activities. All rhizosphere soils from ginseng seedlings were separately collected, mixed, and divided into two parts: one part was stored at 4 °C for analyzing soil physicochemical properties, while the other portion was preserved at −80 °C for subsequent analysis of microbial properties and enzyme activity. Three replicates were prepared for each experiment.

#### 2.2.2 Morphology index characterization of ginseng seedlings

Root length was measured with a Vernier caliper, and plant height was determined with a ruler. Germination percentage was determined on the 14th day using the formula: (number of germinated seeds/total seeds) × 100%.

#### 2.2.3 Resistance characterization of ginseng seedlings

Fresh ginseng seedlings (0.1 g) were homogenized in 0.9 mL phosphate-buffered saline (PBS, 0.05 mol/L, pH 7.4) using an ice bath. Superoxide dismutase (SOD), peroxidase (POD), and catalase (CAT) activities and ginseng soluble sugar (SS) content were measured using commercial assay kits (Nanjing Institute of Bioengineering, Nanjing, China).

### 2.3 Bacterial community characterization

Soil DNA was extracted using the E. Z. N. A.® Soil DNA Kit (Omega Bio-tek, USA), according to the manufacturer’s instructions. Integrity was verified by 1% agarose gel electrophoresis, and concentration (20 ng/μL) and purity (260/280 ratio 1.8–2.0) were detected using a NanoDrop 2000 spectrophotometer (Thermo Fisher Scientific, USA). Primers 338F (5′-ACTCCTACGGGAGGCAGCA-3′) and 806R (5′- GACTACHVGGGTWTCTAAT-3 ′) were used to amplify the bacterial 16S rRNA gene V3-V4 region. The PCR system (50 μL) contained 25 μL of 2 × Premix Taq premix (Takara, China), 1 μL of primers (10 μM), and 3 μL of DNA template. The thermal cycling program was as follows: 95 °C pre-denaturation for 5 min; 30 cycles of 94 °C denaturation for 30 s, 52 °C annealing for 30 s, and 72 °C extension for 30 s; and 72 °C extension for 10 min. The amplified product was screened by agarose gel electrophoresis, and NEBNext® Ultra™ was used. The II DNA library kit (NEB, USA) was used to prepare the library, and double-ended 250 bp sequencing was performed on the Illumina NovaSeq 6,000 platform (Meige Gene, China). After Fastp quality control, Cutadapt primer removal, and USEARCH splicing to remove chimeras from the raw data, 99.9% similarity ASVs were clustered using UPARSE, and species annotation was performed based on the SILVA 138 database. *α*-diversity (Shannon/Chao1) and *β*-diversity [non-metric multidimensional scaling (NMDS)] analyses were performed using QIIME2 and the R language.

### 2.4 Carbon source utilization analysis of microbial communities

The carbon source metabolism activity of the soil microorganisms was determined using the Biolog ECO technique. A fresh sample of soil (10 g) was weighed and placed into a 90 mL 0.85% NaCl aqueous solution in a sterilized triangular flask. The mixture was shaken at 25 °C for 30 min at a speed of 200 rpm and diluted to 0.001 g/mL with 0.85% NaCl solution. Then, 150 μL of diluent was added to 96 wells of the Biolog ECO plate and kept in the dark at 25 °C for 7 days. OD_590_ of the reaction solution was measured every 24 h on a Biolog ECO plate reader.

Average well color development (AWCD) calculation. AWCD characterized the overall color changes in each well and reflected metabolic activity. It was calculated as follows:


$$\text{AWCD}=\frac{\Sigma (\mathrm{Ci}-R)}{n}$$

where Ci is the OD_590_ value of the carbon source compound solution, R is the OD_590_ value of the deionized water, and n is the total number of carbon source compounds. However, if Ci – R ≤ 0, Ci – R = 0.

Utilization rate (Pi) calculation. Pi for each carbon source category was calculated as follows:


$$\mathrm{Pi}=\frac{\mathrm{Ci}-R}{\Sigma (\mathrm{Ci}-R)}$$

Diversity index calculation. The Shannon–Wiener and Simpson indices are expressed as follows:


Shannon−Wiene=−ΣPilnPi;

Simpson=1−Σ(PixPi)

### 2.5 Physicochemical properties and enzymatic activities of the rhizosphere soils

#### 2.5.1 Physicochemical properties characterization of soil

The pH value was measured using a pH meter with a water-to-soil mass ratio of 5:1. Total nitrogen (TN) was determined using the Kjeldahl method. The total phosphorus (TP) content was measured using the NaOH alkali melting molybdenum antimony anti-spectrophotometric method. The total potassium (TK) content was determined using the NaOH melting method. Available nitrogen (AN) was determined using the alkaline hydrolysis diffusion method. Available phosphorus (AP) content was tested by spectrophotometry. Available potassium (AK) was determined using the ammonium acetate leaching method. The organic matter (OM) content was determined using the potassium dichromate volumetric method.

#### 2.5.2 Enzyme activity characterization of soil

Urease (UE), acid phosphatase (ACP), sucrase (SC), and catalase (SCAT) activities were determined using commercial kits (Nanjing Jiang Biologicals) according to the manufacturer’s instructions.

### 2.6 Data statistics and analysis

Basic statistical analysis was performed using one-way analysis of variance (ANOVA) followed by Duncan’s multiple comparison test to assess significant differences among the four treatment groups. Different lowercase letters indicate significant differences at *p* < 0.05. Multivariate statistical analyses included NMDS to evaluate *β*-diversity patterns of microbial communities, linear discriminant analysis effect size (LEfSe) to identify differentially abundant bacterial taxa among groups, principal component analysis (PCA) to examine variations in carbon source utilization profiles of microbial communities, redundancy analysis (RDA) to explore the relationships between soil properties and microbial community factors, and co-occurrence network analysis to analyze interactions among major bacterial taxa. All multivariate statistical visualizations (LEfSe, PCA, NMDS, RDA, and co-occurrence) were conducted using the Wekemo Bioincloud platform^1^. Spearman correlation analysis (SPSS 22.0) was conducted to evaluate associations among plant, soil, and microbial parameters, with |*R*| > 0.6 and *p* < 0.05 defining statistically significant correlations. Line graphs, bar charts, box plots, and heatmaps were generated using GraphPad Prism 9.0 software.

## 3 Result

### 3.1 Characterization of MSN and CS@MSN-SA

**Figure 1:**
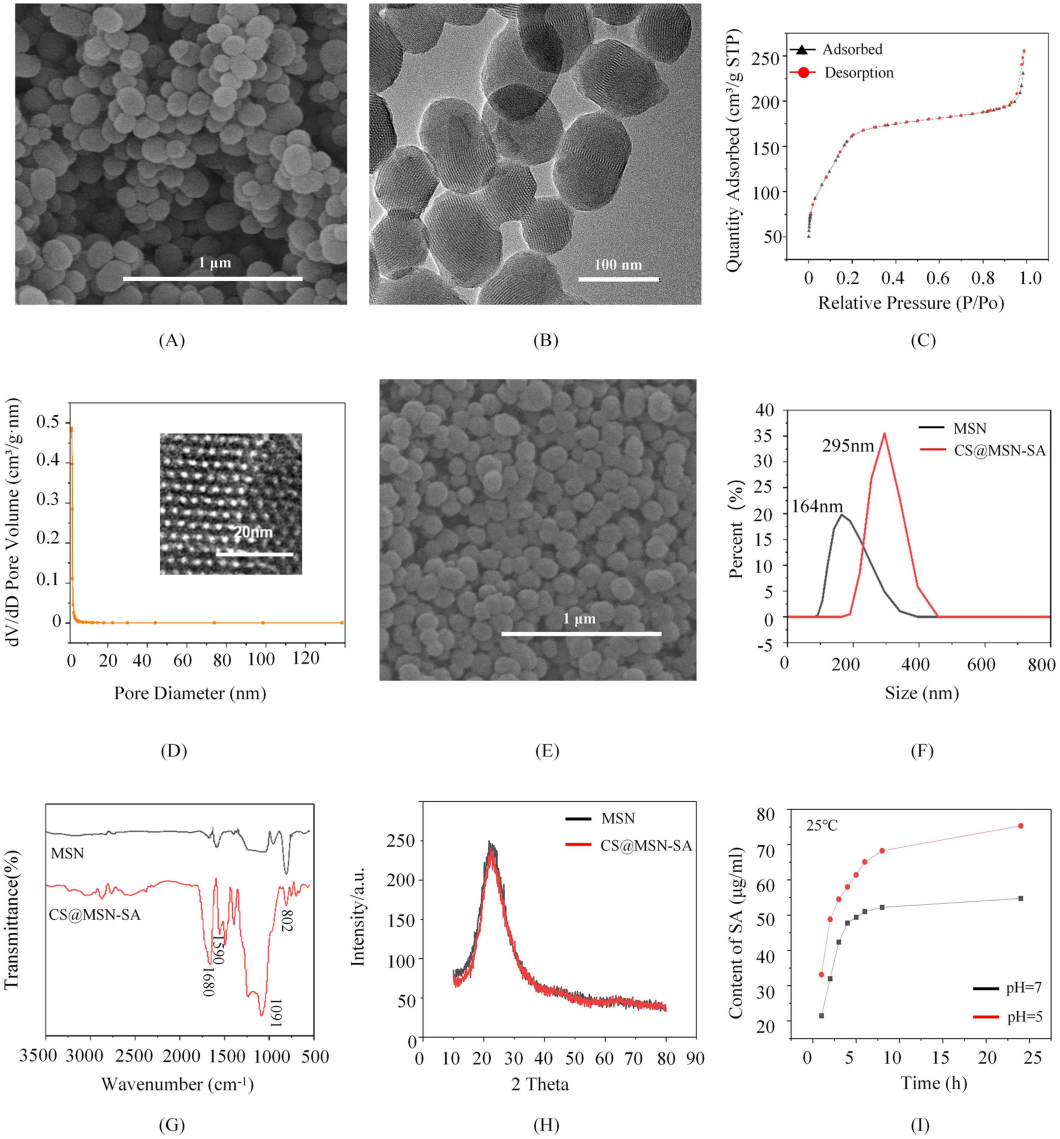
Physicochemical properties of the nanoparticles. **(A)** SEM image, **(B)** TEM image, **(C)** N_2_ adsorption–desorption isotherms curve, and **(D)** pore-size distributions of MSN. **(E)** SEM image of CS@MSN-SA. **(F)** DLS analysis, **(G)** FTIR spectra, and **(H)** XRD patterns of MSN and CS@MSN-SA. **(I)** SA release curves for CS@MSN-SA at different pH values.

MSN displayed a well-defined spherical morphology with mesoporous features, measuring 100–300 nm in diameter, as observed by SEM (Figure 1A). The nanoparticles exhibited a good dispersion without agglomeration. The uniform porous structure of MSNs was clearly shown in TEM imaging, with pore sizes of approximately 2–5 nm (Figure 1B). A typical Type IV isotherm was observed in the N₂ adsorption–desorption analysis of MSN, which is consistent with mesoporous materials (Figure 1C). The value of the BET surface area and Barrett–Joyner–Halenda (BJH) pore size were 2.04963 m^2^/g and 3.3889 nm, respectively, suggesting the excellent drug loading capacity and bioactivity of MSNs (Figure 1D). CS@MSN-SA maintained a spherical structure comparable to MSNs but exhibited increased dimensions, with an average diameter of 300 nm, as confirmed by SEM and DLS (Figures 1E,F). FTIR spectroscopy of MSN (Figure 1G) showed characteristic peaks at 1091 cm^−1^ (asymmetric Si-O-Si stretching) and 802 cm^−1^ (symmetric stretching), confirming the integrity of the silica framework. In the FTIR spectrum of CS@MSN-SA, a broad O-H stretching vibration spanning 3,000–2,500 cm^−1^ and a prominent C=O stretching peak at 1680 cm^−1^ were attributed to the carboxyl and phenolic moieties of SA, confirming the successful loading of SA. The presence of a characteristic CS vibration at 1590 cm^−1^ (N–H in-plane bending of primary amines) confirmed the successful formation of a CS coating. XRD analysis showed that the crystals of CS@MSN-SA and MSN were amorphous, and the modification of CS and SA reduced the diffraction intensity (Figure 1H). The SA loading capacity of CS@MSN-SA was 14%, and the CS encapsulation efficiency was 11.2%, which was obtained by ultrasonic treatment in an aqueous solution for an extended period. Release studies showed that pure SA dissolved completely in water within 45 min, while CS@MSN-SA exhibited sustained release, reaching equilibrium at 24 h. Notably, the release rate at pH 5 was ~25% higher than that at neutral pH (Figure 1I), demonstrating that CS@MSN-SA was pH-responsive, releasing more SA under acidic conditions.

### 3.2 Effect of CS@MSN-SA on the agronomic traits and antioxidant bioactivity of ginseng seedlings

**Figure 2:**
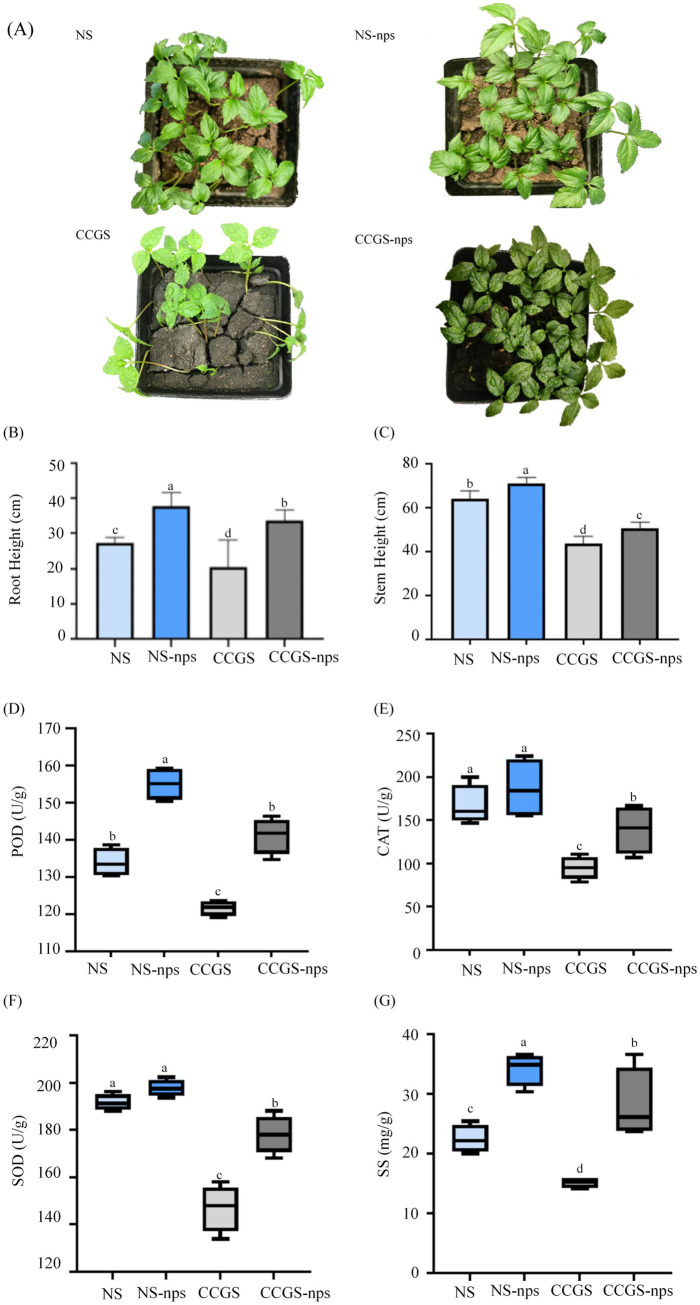
Effect of CS@MSN-SA on the growth of ginseng seedlings. **(A)** Ginseng seedling images. **(B)** Root length, **(C)** Stem height, **(D)** POD activity, **(E)** CAT activity, **(F)** SOD activity, and **(G)** SS content of ginseng seedlings.

To evaluate the effects of CS@MSN-SA on ginseng seed germination, germination rates were recorded for all treatment groups on the 14th day. Germination rates were 91.30 ± 1%, 95.3 ± 3%, 64.70 ± 3%, and 88 ± 2% for NS, NS-nps, CCGS, and CCGS-nps, respectively. The NS group demonstrated higher germination rates compared to the CCGS group, whereas the CS@MSN-SA-treated groups showed superior performance relative to the untreated controls. The effects of CS@MSN-SA on the growth of ginseng seedlings were also investigated (Figure 2A). Ginseng seedlings grown in NS showed a significant growth advantage compared to those grown in CCGS, as evidenced by the root and stem length data (*p <* 0.05). NS-nps treatment significantly enhanced morphological development in ginseng seedlings, exhibiting 40 and 12% greater stem and root elongation, respectively, compared to the NS control. Notably, CCGS-nps demonstrated superior growth-promoting effects with 47 and 40% enhancements in stem height and root length, respectively, relative to CCGS baseline measurements (Figures 2B,C). The growth differences were further reflected in the plants’ stress resistance, as indicated by the antioxidant enzyme activities (Figures 2D–F). For POD, CAT, and SOD activities of ginseng seedlings, there were significant differences between the CCGS and NS groups (*p <* 0.05). After treatment with CS@MSN-SA, POD, CAT, and SOD activities of ginseng seedlings in NS were increased by 15.72, 11, and 3.18%, respectively, while the increases were 16.01, 46, and 21.14%, respectively, for ginseng seedlings in the CCGS group. The SS content in the NS group was higher than that in the CCGS group (*p <* 0.05) (Figure 2G). CS@MSN-SA treatment increased the SS content of ginseng seedlings (*p <* 0.05) in both CCGS and NS. CS@MSN-SA not only promoted agronomic traits but also enhanced the resistance of ginseng seedlings, with more significant effects observed in the CCGS group. Therefore, CS@MSN-SA could promote growth and increase the resistance of ginseng seedlings at the same time.

### 3.3 Effect of CS@MSN-SA on soil bacterial community

**Figure 3:**
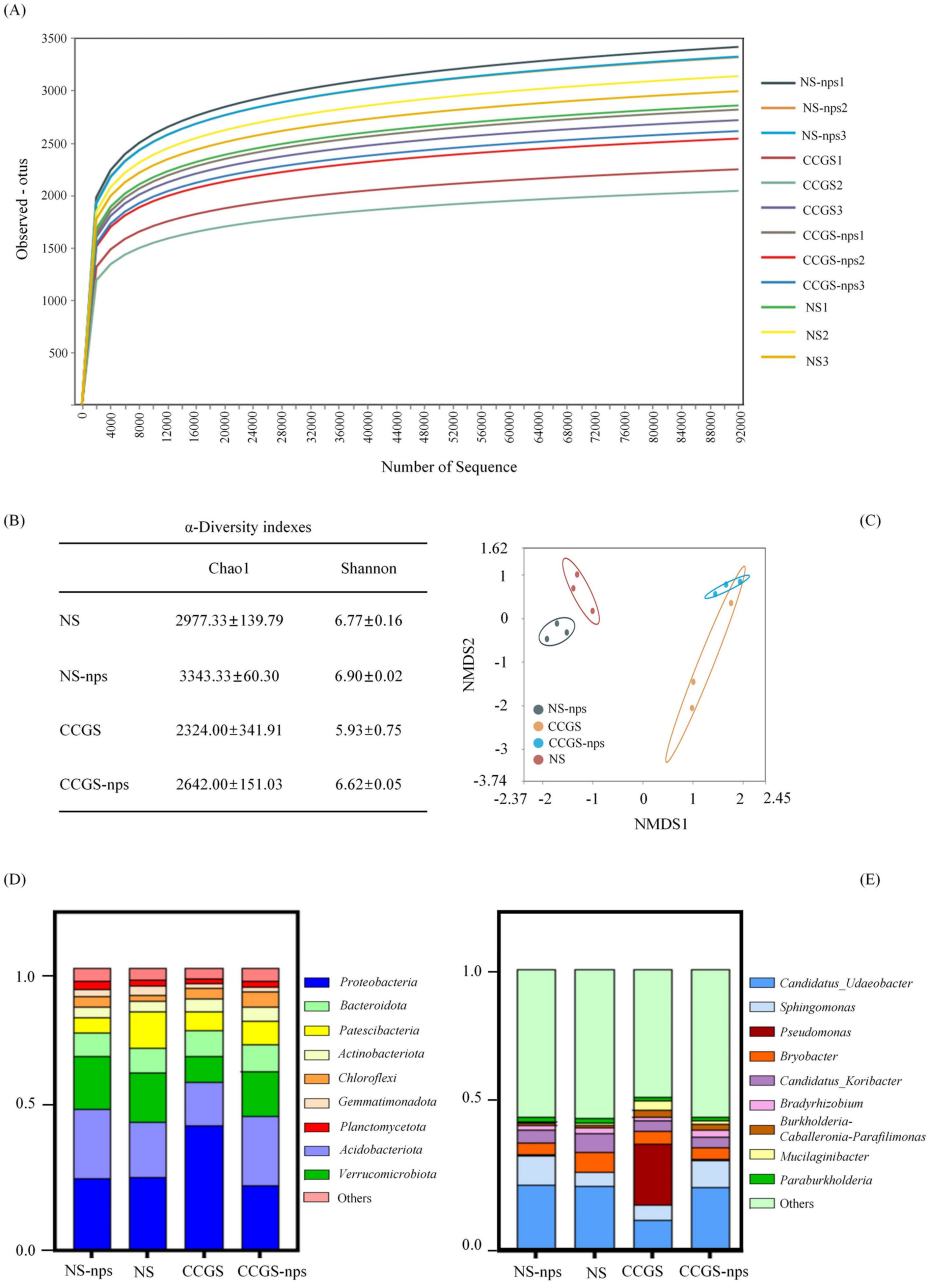
Effect of CS@MSN-SA on the soil bacterial community. **(A)** Dilution curves. **(B)** Chao1 and Shannon indices. **(C)** NMDS analysis of β-diversity. Bacterial community composition at the **(D)** phylum and **(E)** genus levels.

**Figure 4:**
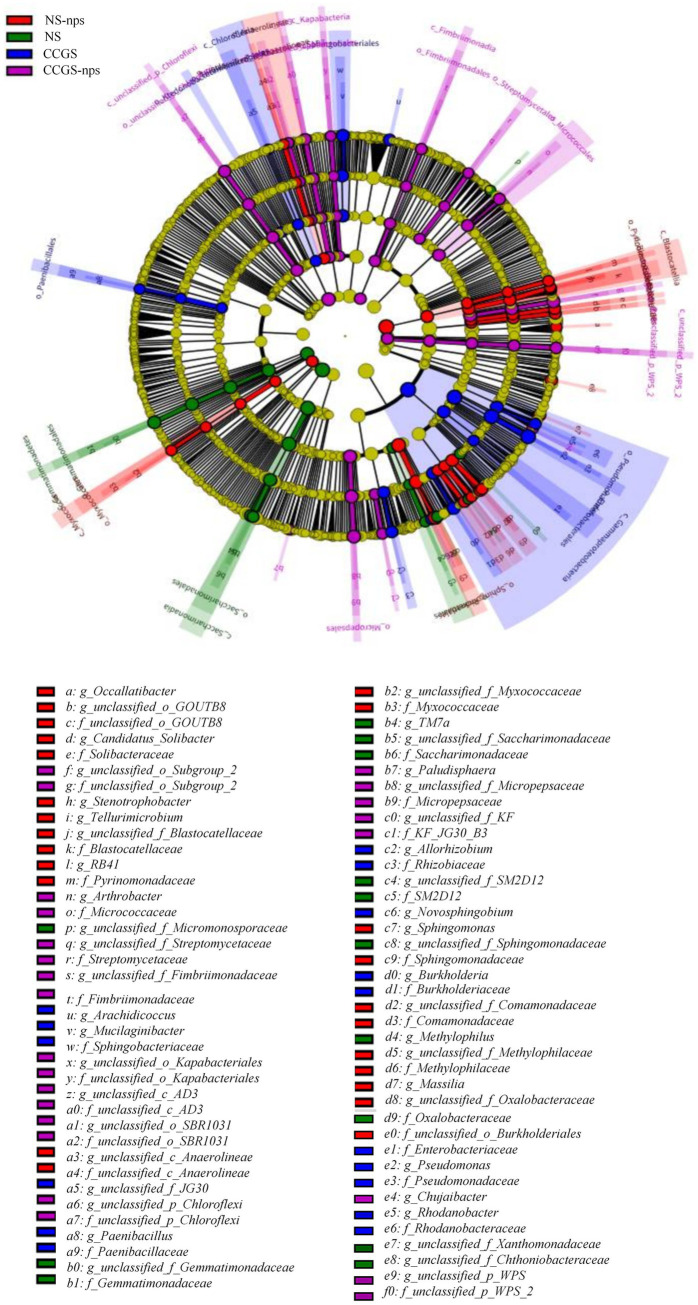
LEfSe analysis of the bacterial community in different soil treatments.

In ginseng cultivation soils, bacterial communities follow successional dynamics correlated with cultivation years, whereas fungal assemblages change stochastically (Fujii et al., 2021). Therefore, composition changes of the bacterial community with different soil treatments were analyzed. The rarefaction curves of the soil samples are shown in Figure 3A. The curve tended to flatten, indicating that the sample quantity was reasonable and the sequencing depth was large enough for bacterial diversity analysis. Compared to the CCGS group, *α*-diversity analysis showed a 28.1% higher Chao1 index in the NS group with the CS@MSN-SA amendment, increasing the species richness by 12–14% across treatments (Figure 3B). Additionally, Shannon diversity patterns mirrored these trends, confirming the efficacy of CS@MSN-SA in mitigating CCGS-induced biodiversity loss. NMDS revealed significant treatment-specific clustering, where CCGS-nps formed transitional states between the CCGS and NS groups, suggesting partial restoration of microbial architecture toward healthy profiles (Figure 3C). The main phyla (relative abundance ≥ 1%) were *Proteobacteria*, *Acidobacteriota*, *Verrucomicrobiota*, *Bacteroidota*, *Patescibacteria*, *Actinobacteriota*, *Planctomycota*, *Gemmatimonadota*, and *Chloroflexi*. The sum of these phyla accounted for 95% of the total bacteria. Compared to the NS group, the relative abundance of *Chloroflexi* and *Proteobacteria* increased in the CCGS group, while the relative abundance of *Verrucomicrobiota*, *Patescibacteria*, and *Gemmatimonadota* decreased (Figure 3D). CS@MSN-SA treatment mitigated these changes in the CCGS group. Compared with the NS group, the regulatory role of CS@MSN-SA in the health community in the NS-nps group was not obvious. The top nine genera (relative abundance ≥1%) were *Candidatus_Udaeobacter*, *Sphingomonas*, *Pseudomonas, Bryobacter*, *Candidatus_Koribacter*, *Bradyrhizobium*, *Burkholderia-Caballeronia-Paraburkholderia*, *Mucilaginibacter*, and *Parafilimonas* (Figure 3E). The relative abundances of *Candidatus_Udaeobacter*, *Sphingomonas*, and *Bradyrhizobium* were higher in the NS group than in the CCGS group, whereas the relative abundances of Pseudomonas and Mucilaginibacter were lower. CS@MSN-SA treatment mitigated these changes in the CCGS-nps group. Compared with the NS group, CS@MSN-SA treatment upregulated the abundance of *Sphingomonas* and *Parafilimonas* and downregulated the abundance of *Mucilaginibacter* and *Candidatus Koribacter* in NS-nps. Inter-group variations in microbial community composition were systematically evaluated using the LEfSe methodology (Figure 4). Bacterial taxa demonstrated significant discriminatory power (LDA score >3) across taxonomic hierarchies and were subsequently identified. The analysis revealed 77 differentially abundant bacterial taxa, comprising 32 family- and 45 genus-level classifications. In the NS treatment group, we detected four distinct families and nine genera, with notable enrichment of beneficial taxa, including *f_Xanthomonadaceae*, *f_Gemmatimonadaceae*, and *f_Sphingomonadaceae*. The NS-nps group exhibited greater microbial diversity, containing 10 family- and 13 genus-level discriminators, particularly *f_Sphingomonadaceae* and *f_Comamonadaceae*, and functionally significant genera, *g_Sphingomonas*, *g_Candidatus Solibacter*, *g_RB41*, and *g_Massilia*. Comparative analysis of the CCGS group demonstrated selective enrichment of seven families and nine genera, with *f_Paenibacillaceae*, *f_Enterobacteriaceae*, *f_Burkholderiaceae*, and genera *g_Pseudomonas*, *g_Mucilaginibacter*, and *g_Arachidicoccus* showing predominant LDA values. The CCGS-nps treatment group presented the most complex microbial profile, containing 11 family- and 13 genus-level biomarkers, including ecologically significant unclassified *Chloroflexi* taxa at both the genus (*g_unclassified_p_Chloroflexi*) and family (*f_unclassified_p_Chloroflexi*) levels, along with *g_Arthrobacter*, a genus recognized for its bioremediation potential. This hierarchical analysis revealed treatment-specific microbial signatures, with nanoparticle-supplemented groups demonstrating enhanced proliferation of functionally advantageous taxa compared to their respective baseline treatments.

### 3.4 The effect of CS@MSN-SA on the carbon source utilization of the soil microbial community

**Figure 5:**
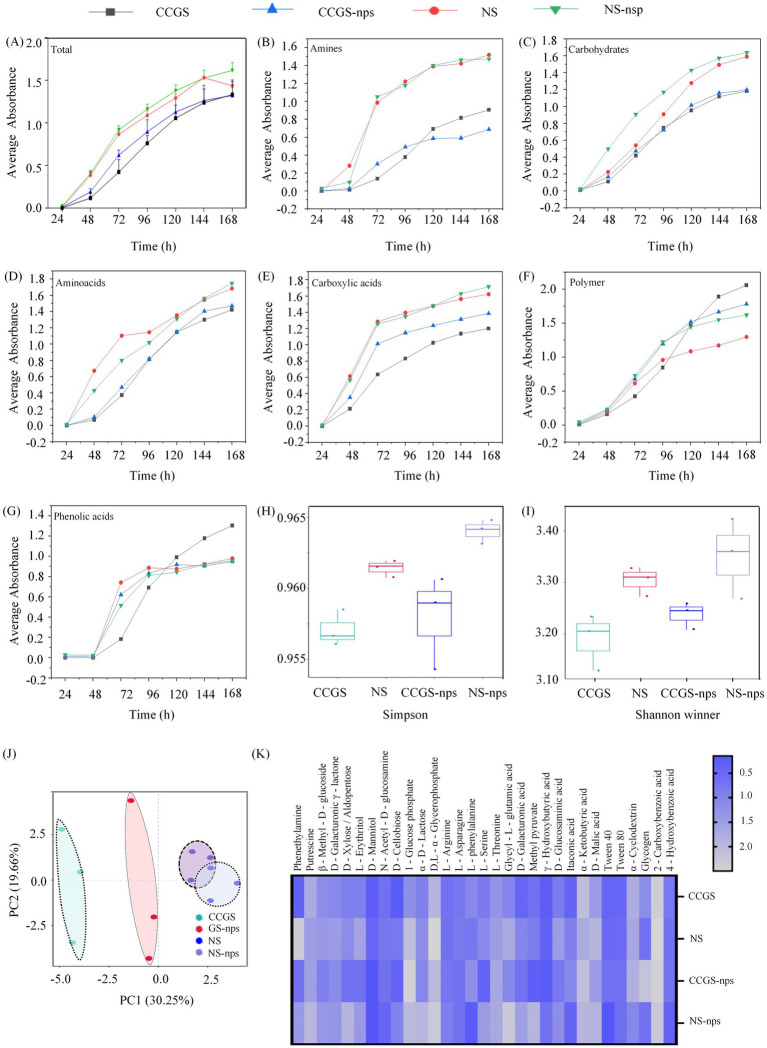
Effects of CS@MSN-SA on microbial carbon source utilization. AWCD of **(A)** Total carbon sources, **(B)** amines, **(C)** carbohydrates, **(D)** amino acids, **(E)** carboxylic acids, **(F)** polymers, and **(G)** phenolic acids. **(H)** Simpson’s index. **(I)** Shannon index. **(J)** PCA of carbon metabolism. **(K)** Heat map of the utilization of the 31 carbon substrates. **(H–K)** Microbial metabolic profiles based on Biolog ECO microplate assays after 144 h of incubation.

The metabolic functional potential of soil microbial communities across experimental treatments was quantitatively assessed through temporal monitoring of AWCD values using 31 ecologically relevant carbon substrates. Figure 5A demonstrates significantly higher baseline metabolic activity in NS compared to CCGS counterparts (*p* < 0.05). Notably, the CS@MSN-SA amendment enhanced the metabolic potential in nanoparticle-free soil systems. Figures 5B–G revealed time-dependent metabolic specialization patterns across carbon substrate categories. During the initial 96 h, the NS soil microbiota demonstrated significantly accelerated substrate utilization kinetics compared to CCGS communities across all six substrate classes: amines, amino acids, carboxylic acids, carbohydrates, polymers, and phenolic acids. CS@MSN-SA amendment induced differential metabolic reprogramming: in CCGS soils, it enhanced all substrate catabolism, while in NS systems, it selectively upregulated all substrate classes except phenolic acids. Subsequent phase analysis (96–168 h) revealed native CCGS communities’ superior polymer and phenolic acid utilization efficiency, which CS@MSN-SA treatment strategically modulated through coordinated suppression of these pathways with concurrent induction of amine and carboxylic acid metabolism. CS@MSN-SA enhanced carbohydrate and polymer metabolism in the microbial communities of the NS group. AWCD kinetic profiles plateaued at 144 h, signaling microbial metabolic equilibrium establishment across treatment regimes. Comparative analysis of substrate utilization patterns (Figures 5H,I) revealed that NS soils maintained superior microbial metabolic diversity, as evidenced by significantly higher Shannon–Wiener and Simpson indices. CS@MSN-SA amendment universally enhanced diversity metrics across soil types. PCA of terminal-phase metabolism (144 h) resolved 49.91% cumulative variance (PC1:30.25%, PC2:19.66%), identifying distinct biochemical drivers: Glycyl-L-glutamic acid and D-xylose dominated PC1, while D-galacturonic acid and putrescine primarily shaped PC2 (Figure 5J). Intriguingly, L-serine exhibited a dual-axis influence, emerging as a critical metabolic differentiator. Heatmap analysis (Figure 5K) quantified CS@MSN-SA’s dual functionality: restoring the CCO-compromised metabolic pathways and enhancing native soil metabolism. CS@MSN-SA demonstrated unprecedented bifunctional remediation capacity.

### 3.5 Effect of CS@MSN-SA on the physicochemical properties and enzyme activity of soil

**Figure 6:**
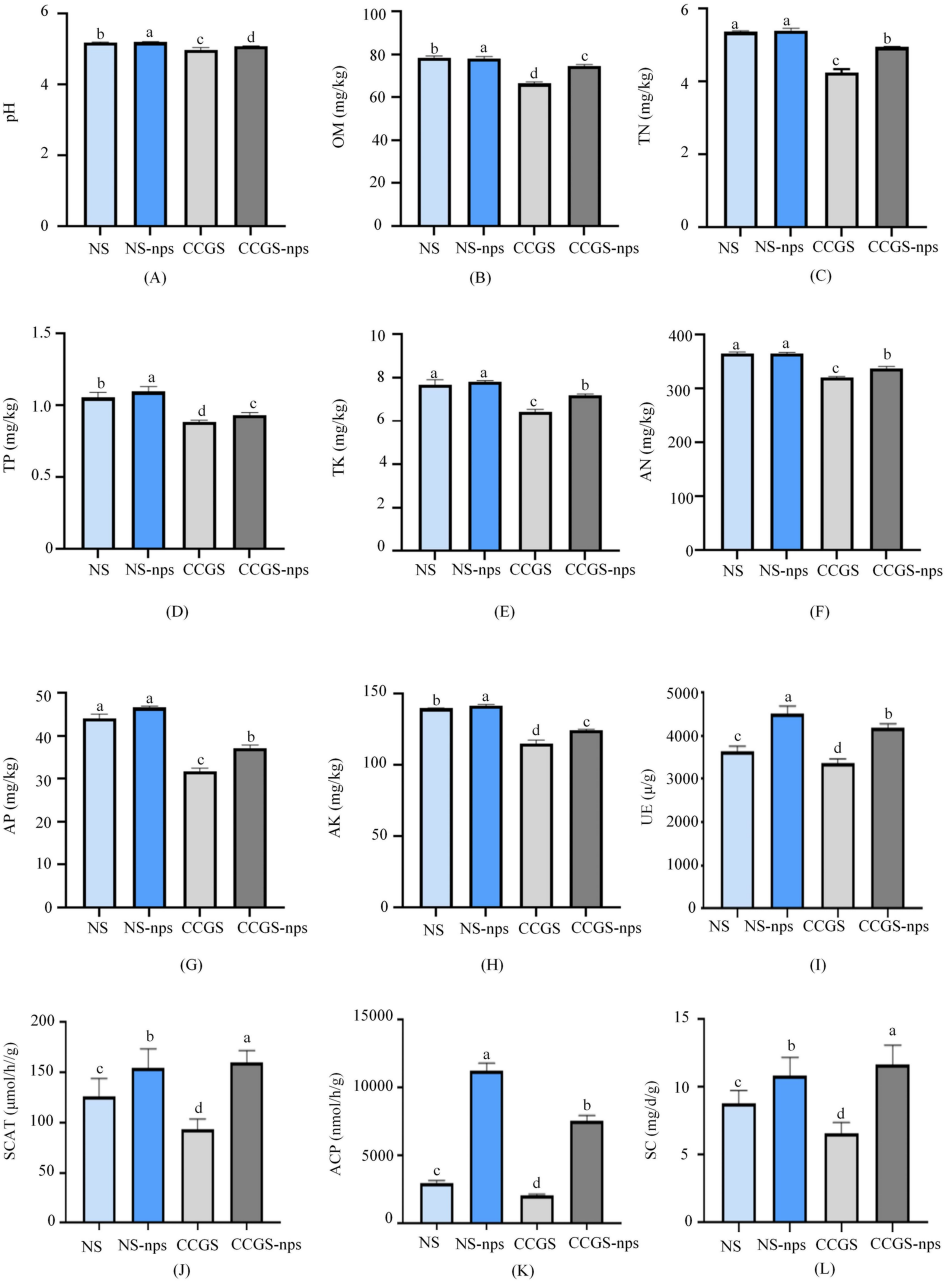
Effect of CS@MSN-SA on soil factors. **(A)** pH, **(B)** OM, **(C)** TN, **(D)** TP, **(E)** TK, **(F)** AN, **(G)** AP, **(H)** AK, **(I)** UE, **(J)** SCAT, **(K)** ACP, and **(L)** SC. Different lowercase letters indicate significant differences at *p* < 0.05.

To investigate the effect of CS@MSN-SA on the physicochemical properties of the soil, eight indices, including pH, OM, TK, TN, TP, AN, AP, and AK, were determined (Figures 6A–H). All eight index levels showed a significant difference between the NS and CCGS groups (*p* < 0.05). After the application of CS@MSN-SA, the levels of the eight indices in the CCGS significantly increased (*p* < 0.05). Compared to the CCGS group, the levels of pH, OM, TK, TN, TP, AN, AP, and AK in the CCGS-nps group increased by 2.15, 5.27, 8.51, 5.95, 5.49, 4.58, 7.55, and 2.05%, respectively. The regulatory roles of CS@MSN-SA on TK and AP content were the strongest among the eight indices. Compared to the NS group, the levels of pH, OM, TK, TN, TP, AN, AP, and AK increased by 2.38, 4.74, 3.28, 2.98, 4.96, 1.03, 2.23, and 3.72%, respectively, in the NS-nps group. After the application of CS@MSN-SA, TP, and AK, levels of NS were significantly promoted (*p* < 0.05), while other index levels only showed an upward trend.

UE, ACP, SCAT, and SC activities were determined to survey the effect of CS@MSN-SA on the bioactivity of the soil (Figures 6I–L). The four index levels in the NS group showed a significant difference compared to the CCGS group (*p* < 0.05). After the application of CS@MSN-SA, the UE, ACP, SCAT, and SC of the soil were significantly elevated (*p* < 0.05). The UE, ACP, SCAT, and SC activities of the CCGS group increased by 27.83, 261.02, 72.62, and 75.95%, respectively, after treatment with CS@MSN-SA; those of the NS group increased by 28.03, 272.47, 21.82, and 23.74%, respectively, after treatment with CS@MSN-SA. The increase in ACP activity was the most remarkable among the four indices, indicating that CS@MSN-SA could regulate the transformation of P. Results of the physicochemical properties and enzyme activity of soil confirmed that CS@MSN-SA significantly influenced the transformation and degradation of P and K elements, increased soil fertility, and decreased the acidity of soils. CS@MSN-SA has the potential to be used as a soil amendment to alleviate the CCO of ginseng.

### 3.6 Correlation analysis between soil and microbial community factors

**Figure 7:**
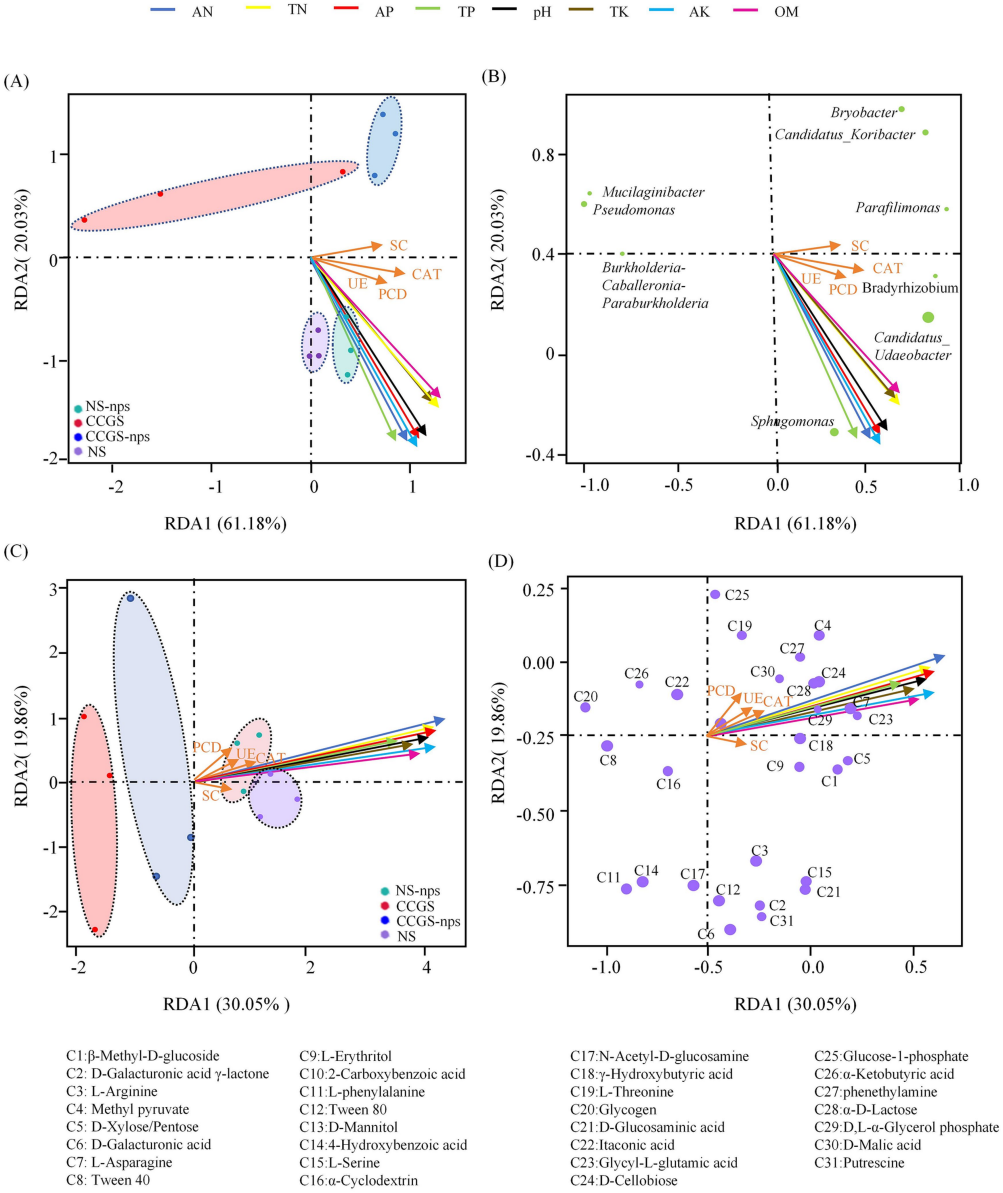
Correlation analysis between soil and microbial community factors. **(A,B)** RDA of soil bacterial community composition with soil factors. **(C,D)** RDA of soil carbon source utilization with soil factors.

Redundancy analysis demonstrated that physicochemical parameters of the soil collectively accounted for 81.21% of microbial community variance (RDA1:61.18%, RDA2:20.03%; *p* = 0.001), with OM and TN dominating primary axis variation, while AP and AK governed secondary differentiation, and 12 soil samples from the four groups were clearly separated (Figures 7A,B). CS@MSN-SA amendment significantly restructured the microbial communities through hierarchical environmental controls. Nutrient-sensitive taxa, including *Sphingomonas* and *Bradyrhizobium,* showed strong positive correlations with AN and AP, whereas *Sphingomonas* and *Bradyrhizobium* showed strong positive correlations with pH (Figure 7C). *Candidatus_Udaeobacter* abundance was driven by AN, AP, TK, and TP. RDA of carbon utilization patterns demonstrated clear group separation (permutation *p* = 0.005), with RDA1 (30.05%) and RDA2 (19.86%) cumulatively explaining 49.91% of the variance (Figures 7D,E). CS@MSN-SA treatment shifted the metabolic drivers, amplifying physicochemical dominance over enzymatic regulation. OM, AN, and AK emerged as the primary determinants of carbon substrate utilization variance. Methyl pyruvate, L-threonine, and glucose-1-phosphate were regulated by OM, AN, and AP. D-Galacturonic acid *γ*-lactone, D-Galacturonic acid, and 4-Hydroxybenzoic acid were driven by pH, TK, and SC. This tripartite regulation highlights physicochemical parameters as master regulators of microbial community architecture and functional plasticity in agricultural systems.

### 3.7 Main mechanism analysis of CS@MSN-SA regulating the growth of ginseng

**Figure 8:**
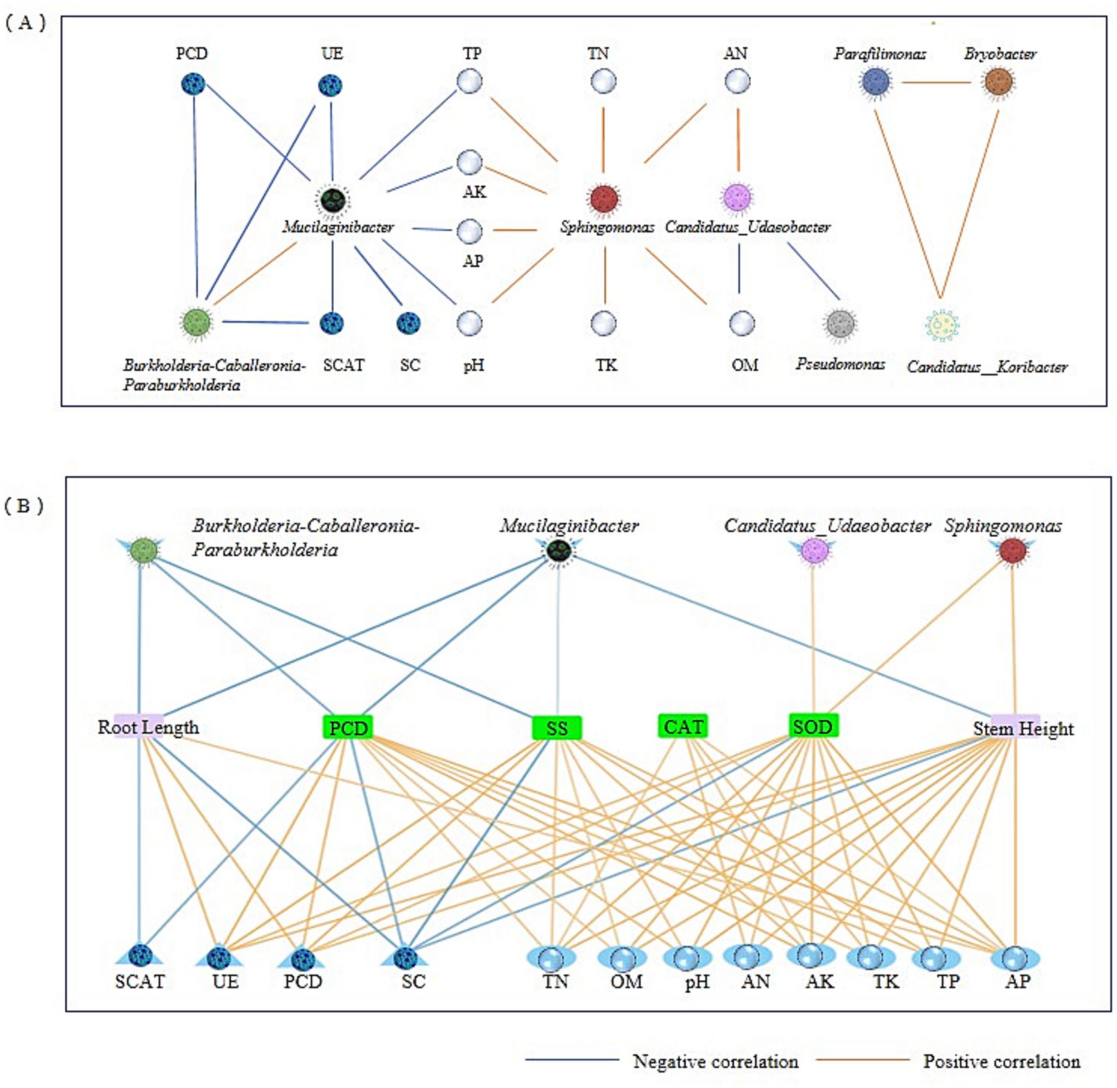
Correlation analysis of soil, ginseng, and microbial community. **(A)** Correlation analysis of the main soil bacterial taxa and soil factors. **(B)** Correlation analysis of the growth indices of ginseng seedlings with soil factors and the main soil bacterial composition.

CS@MSN-SA treatment significantly increased the relative abundance of the microbiota, including *Candidatus_Udaeobacter*, *Sphingomonas*, *Parafilimonas*, and *Bradyrhizobium*, while suppressing the genera *Mucilaginibacter*, *Burkholderia-Caballeronia-Paraburkholderia*, and *Pseudomonas* (Figure 8A). This intervention concurrently enhanced soil physicochemical properties (pH and nutrient availability) and biological activities (UE, CAT, ACP, and SC). In Figure 8B, the correlation between soil, ginseng, and the microbial community was analyzed. *Candidatus_Udaeobacter* demonstrated comprehensive positive correlations with all soil parameters (*p* < 0.05), plant growth promotion, and resistance enhancement. Conversely, *Mucilaginibacter* and *Pseudomonas* exhibited negative regulatory effects. Notably, *Burkholderia-Caballeronia-Paraburkholderia* abundance was inversely correlated with soil enzyme activity, plant POD activity, and SS content (*R* = −0.68). *Parafilimonas* counteracts these effects through positive regulation. *Sphingomonas* showed a universal enhancement of soil physicochemical indicators and plant stress resistance. *Bradyrhizobium* improved most soil parameters, except UE, ACP, and AP, whereas plant CAT and SOD activities improved (*p* < 0.05). All soil parameters were positively correlated with plant resistance indices. Root length showed significant positive associations with all soil metrics, except SC. Similarly, stem length was correlated positively with all parameters, except SC and CAT. These findings demonstrate that CS@MSN-SA promoted the growth of ginseng seedlings, improved soil fertility, and targeted microbial modulation. CS@MSN-SA optimized soil–plant-microbial interactions.

### 3.8 Mechanistic analysis of CS@MSN-SA alleviating CCO

**Figure 9:**
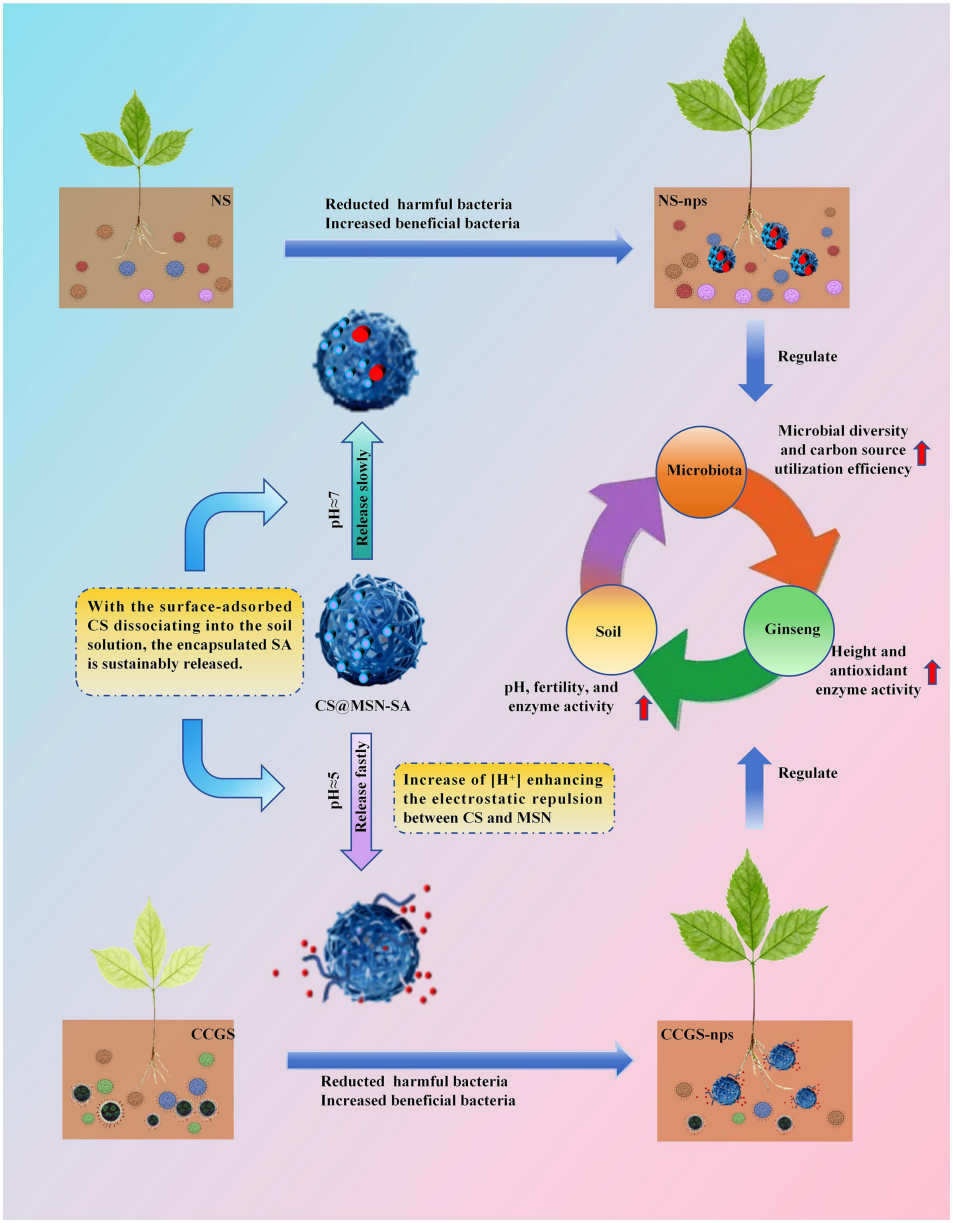
Mechanistic analysis of CS@MSN-SA mitigating CCO.

The mechanistic model of CS@MSN-SA alleviating the CCO of ginseng is shown in Figure 9. CCO refers to the growth inhibition of replanted ginseng, deterioration of soil properties, and imbalance in soil microbial communities. The CS@MSN-SA amendment significantly increased the abundance of beneficial microorganisms, such as *Candidatus_Udaeobacter*, *Sphingomonas*, *Parafilimonas*, and *Bradyrhizobium*, which contributed to nutrient cycling and pathogen suppression. Concurrently, it reduces the abundance of detrimental genera, including *Mucilaginibacter* and *Pseudomonas*, which are often associated with disease incidence in CCO systems. CS@MSN-SA treatment redirected microbial metabolic activities toward the utilization of amines, carbohydrates, and carboxylic acids. Restructured microbiota enhanced enzyme activities (UE, CAT, ACP, and SC), accelerated nutrient cycling, and increased available N, P, K, and OM, thereby improving soil fertility and reducing acidity. These synergistic improvements in soil microbiology and properties promote seed germination, growth, and resistance of ginseng. CS@MSN-SA is a pH-responsive intelligent material. Under neutral soil conditions, SA was slowly released from the nanoparticles via desorption from CS and MSN, and minimal SA release promoted plant growth via hormonal effects. In acidic environments, enhanced repulsion between CS and MSN induced nanoparticle swelling, leading to rapid SA release. SA release promotes plant growth via resistance improvement. This was consistent with greater resistance improvement in CCGS than NS after the treatment of CS@MSN-SA. Therefore, CS@MSN-SA alleviated CCO formation from the initial planting, not only in the replanting phases of ginseng cultivation. The repair function of CS@MSN-SA automatically changed with soil acidity. In summary, CS@MSN-SA acted as a multi-target mediator that systematically integrated soil–plant–microbe interactions, effectively disrupting the negative feedback loop of CCO and offering a sustainable strategy for ginseng cultivation.

## 4 Discussion

This study designed and synthesized a novel soil amendment, CS@MSN-SA, exhibiting multifunctional capacities: (1) restructuring soil microbial communities, enriching beneficial taxa while suppressing pathogenic genera; (2) modulating soil pH toward neutralization and optimizing rhizosphere nutrient cycling; and (3) improving the interactions of soil–plant–microbe. The amendment significantly improved ginseng growth performance in both pristine soils and continuous-cropping-challenged environments, thereby alleviating CCO formation.

Continuous cropping obstacles significantly reduced soil bacterial diversity, particularly depleting *Verrucomicrobiota*, *Patescibacteria*, and *Gemmatimonadota,* while elevating *Proteobacteria* abundance (Xiao et al., 2015). This selective pressure preferentially enriched *Proteobacteria* over *Acidobacteriota* and *Firmicutes*, driving *Pseudomonas* dominance, which was directly correlated with plant growth suppression. Functionally, the *Verrucomicrobiota* were identified as nitrogen-fixing oligotrophs (Fang et al., 2024), whereas both *Patescibacteria* and *Verrucomicrobiota* showed significant positive correlations with AP (Fang et al., 2025). *Gemmatimonadota* has multifunctional roles in phosphorus cycling, exhibiting positive associations with TN, AP, AK, pH, and OM (Gui et al., 2024). CCO significantly reduced the relative abundance of beneficial taxa, including *Candidatus_Udaeobacter*, *Sphingomonas*, and *Bradyrhizobium*, while concurrently enriching the genera *Pseudomonas* and *Mucilaginibacter*. LEfSe analysis identified *Sphingomonadaceae*, *Comamonadaceae*, and *Xanthomonadaceae* as key discriminators for NS soil, whereas *Paenibacillaceae*, *Enterobacteriaceae*, *Burkholderiaceae*, *Pseudomonas*, *Mucilaginibacter*, and *Arachidicoccus* characterized CCGS communities. *Candidatus_Udaeobacter* demonstrated multifunctional carbon metabolism (glucose, pyruvate, and chitobiose) and lytic activity against antibiotic-producing bacterial populations. *Sphingomonas* exhibited xenobiotic degradation capacity for diphenylether and isoproturon (Li S. et al., 2024), whereas *Bradyrhizobium* is positively correlated with ginseng biomass and saponin content (Shi et al., 2024). Conversely, *Pseudomonas* dominance showed negative phytochemical correlations with plants (Xiao et al., 2015). Notably, NS-enriched *Xanthomonadaceae* and *Comamonadaceae* displayed nitrogen fixation and fusarium toxin degradation (Urbaniak et al., 2020). *Sphingomonadaceae* exhibited phosphate solubilization, indole-3-acetic acid production, and aromatic compound catabolism (Cho et al., 2024). CCGS-specific *Paenibacillaceae* and *Enterobacteriaceae* demonstrated *β*-glucosidase activity, enabling ginsenoside deglycosylation (Zhao et al., 2016; Siddiqi et al., 2017a; Kim et al., 2009; Siddiqi et al., 2017b; Cui et al., 2017) and structurally amplifying allelopathic effects (Zhou et al., 2024). This microbial shift in the CCGS group exacerbated allelochemical stress while impairing nitrogen fixation. Comparative studies revealed consistent temporal patterns: *Burkholderiaceae* and *Enterobacteriaceae* enrichment and *Sphingomonas* depletion with cultivation duration (Li K. et al., 2024; Dong et al., 2018) and were supported by genus-level antagonism (*Sphingomonas* vs. *Burkholderia*; *Pseudomonas* vs. *Xanthomonas*) (Barone et al., 2024).

The CS@MSN-SA amendment significantly increased the relative abundance of beneficial taxa, including *Candidatus_Udaeobacter*, *Bryobacter*, and *Bradyrhizobium,* while suppressing *Mucilaginibacter*. LEfSe analysis identified unclassified members in the *Chloroflexi* phylum and *SBR1031* order and *the Arthrobacter* genus as key discriminators for CCGS-nps soils. Functionally, *Bryobacter* has demonstrated antifungal activity against the ginseng root pathogen, *Fusarium*. Chloroflexi species showed significant positive correlations with soil conductivity, CAT, UE activity, TN, AP, AK, pH, and OM content (Gui et al., 2024). *SBR1031* enhanced microbial network complexity through ecological niche occupation (You et al., 2025), whereas *Arthrobacter nicotinovorans JI39* upregulated phenylpropanoid biosynthesis while enhancing plant SOD, POD, and UE activities (Jiang et al., 2022). The amendment enhanced plant stress resistance through multiple mechanisms, including ecological niche optimization for beneficial microbiota, pathogen-resistant bacterial enrichment (*Bacillus*), suppression of saponin-degrading bacteria (*Mucilaginibacter*), and soil quality improvement. These microbial shifts aligned with the patterns observed in corn biochar-amended soils (Liu et al., 2022).

In the NS control soil, CS@MSN-SA treatment increased *Sphingomonas* abundance while reducing *Mucilaginibacter* and *Candidatus Koribacter*. The *F_Sphingomonadaceae* and *F_Comamonadaceae* families, along with the *Sphingomonas*, *Candidatus Solibacter*, *RB41*, and *Massilia* genera, emerged as significant contributors to NS-nps communities. *RB41* functioned as a crucial regulator of nutrient flux dynamics through enhanced functional redundancy mechanisms, whereas *Massilia* demonstrated potent phosphate solubilization capacity, potentially enhancing phosphorus bioavailability (Xu et al., 2022). The high LDA score of *Candidatus Solibacter* suggested accelerated chitin coating degradation on nanoparticles, providing supplemental carbon sources for microbial communities. In the initial planting of ginseng seedlings, CS@MSN-SA played a role in accelerating the nutrient cycle.

CCGS exhibited significantly reduced metabolic activity compared to NS. CCGS specifically suppressed amine metabolism while enhancing polymer and phenolic acid utilization. Elevated phenolic acid metabolism might be associated with root-secreted phenolic compound accumulation in CCGS, whereas polymer utilization likely originated from plant residue accumulation in aged ginseng fields. The decrease in the utilization rate of amine carbon sources was related to the inhibition of UE activity in CCGS. The CS@MSN-SA amendment increased carboxylic acid metabolism by 14.52%, while reducing phenolic acid utilization by 24.05% in CCGS-nps, corresponding to decreased root phenolic secretions and enhanced plant growth demands. Organic acids are removed from the secretion point of the root more quickly and are more easily utilized by microbial communities than carbohydrate carbon sources (Wiesenbauer et al., 2025). In the NS-nps group, phenolic acid utilization decreased by 6.0%, and amines and carboxylic acids were slightly enhanced compared to those in the NS group, suggesting metabolic rebalancing of growth-promoting substrates. CS@MSN-SA optimized the microbial prioritization of carboxylic acids over phytotoxic phenolic compounds following soil remediation.

**Figure 10:**
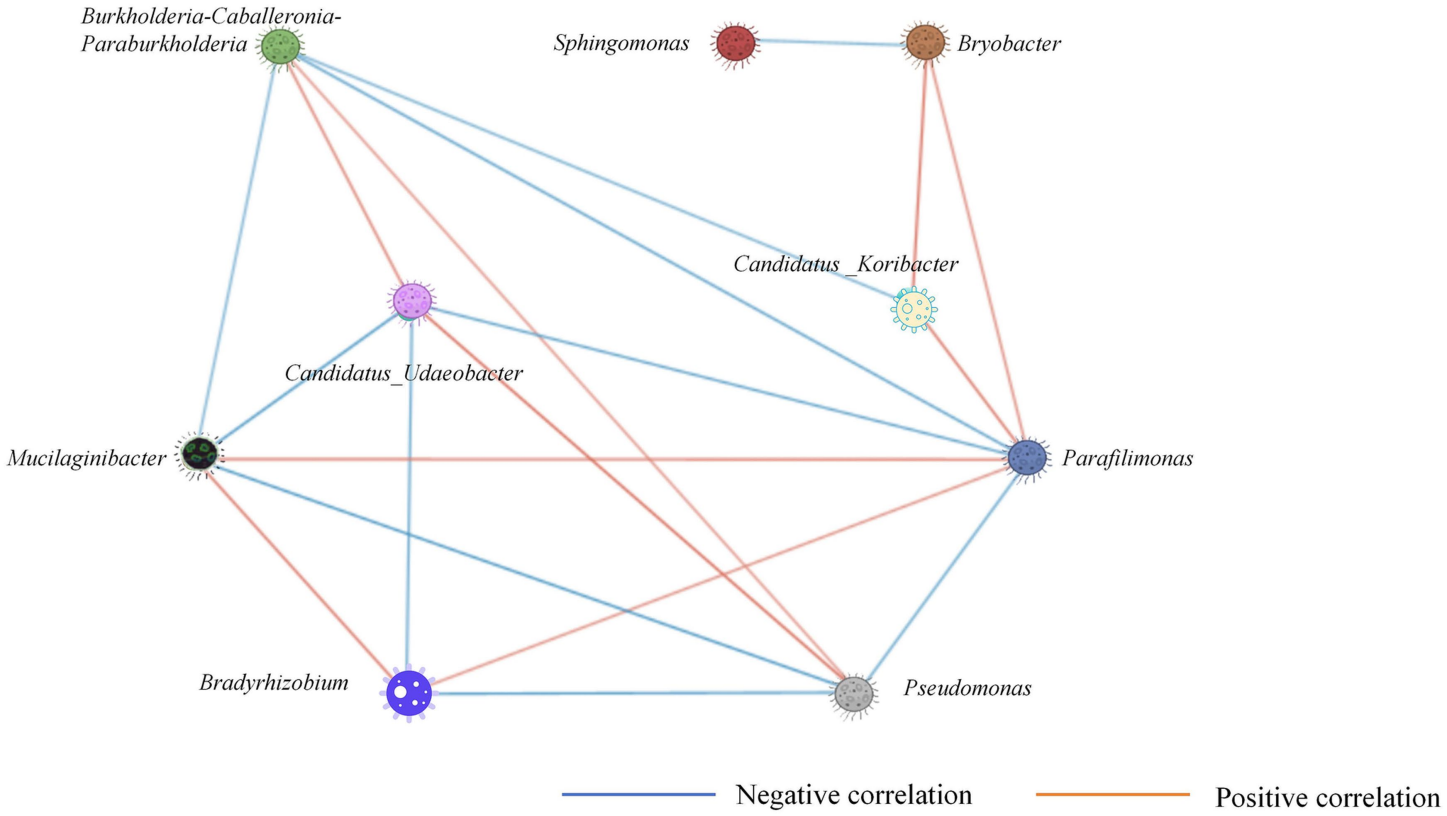
Co-occurrence network analysis of the main soil bacterial taxa.

Soil–microbe interactions constituted a multidimensional interplay where microbial communities profoundly modulated soil physicochemical properties, nutrient cycling dynamics, and enzymatic activities, while soil factors reciprocally governed microbial distribution and consortia composition. This system features an intricate network of symbiosis and niche competition within the microbial consortia, coupled with synergistic or antagonistic interdependencies among environmental parameters. Functional profiling revealed the nitrogen-fixing, phosphate-solubilizing, and nutrient-recycling capacities of key taxa, suggesting tight biogeochemical coupling. Spearman’s correlation analysis demonstrated significant associations between the dominant microbial taxa (Figure 10). RDA identified soil factors as regulators of core genera. Strong positive correlations (*R* > 0.7) were observed among the soil factors. Therefore, CS@MSN-SA promoted growth and resistance through strategic network modulation of soil microbes. CS@MSN-SA alleviates CCO by optimizing the soil–plant-microbial cycle.

The potential ecological risks of SiO₂ nanoparticles (NPs) to soil and crop safety have attracted widespread attention in recent years. Extensive studies have indicated that low concentrations of SiO₂ NPs could alleviate plant abiotic stress and suppress soil degradation without exhibiting ecotoxicity (Schaller et al., 2025). However, at high concentrations and small particle sizes, SiO₂ NPs may exert some effects on the soil and plants. For instance, spherical SiO₂ NPs with a particle size of approximately 15 nm can induce avoidance behavior in *Enchytraeus crypticus* and *Eisenia fetida* at soil concentrations exceeding 500 mg/kg without significantly impairing soil habitat function (Santos et al., 2020). The addition of 1–2% SiO₂ NPs effectively reinforced the structure of clay and sandy soils, which may impose mechanical resistance to plant root elongation (Gu et al., 2022). SiO₂ NPs, approximately 30 nm in diameter, inhibited the growth of Bt-transgenic cotton in a concentration-dependent manner, suppressing morphological traits, antioxidant capacity, nutrient uptake, and endogenous hormone levels (Le et al., 2014). The toxicity intensity was correlated with the crystallinity, size, dispersion, and concentration of silica, although the overall toxicity of the SiO₂ NPs remained weak. Some studies confirm that even at concentrations as high as 1,000 ppm, SiO₂ NPs exhibited no significant phytotoxicity. In buffered soil environments, the associated toxicity risks are low (Slomberg and Schoenfisch, 2012). The synthesized MSN in this study had a particle size of approximately 300 nm with an amorphous crystal structure and was surface-modified with CS and SA molecules. Both modifying agents were naturally derived compounds with good biocompatibility and could be biodegraded by soil microbes, posing no ecological hazard to the soil. The nanoparticles were applied at a concentration of 0.83 mg/g, which is below 1% and unlikely to impede ginseng root growth. Controlled experiments confirmed that there were no adverse effects on the soil microbial communities or plant physiological indicators. Owing to time limitations, the long-term toxicity of the nanoparticles was not examined in this experiment but will be thoroughly investigated in future studies.

## 5 Conclusion

In this study, a pH-responsive intelligent soil amendment, CS@MSN-SA, was designed and synthesized. CS@MSN-SA realized the controlled release of SA effectively in different pH environments and promoted seed germination and seedling growth of ginseng by reconstructing the microbe, enhancing soil fertility, and modulating microbial community-soil interactions. CS@MSN-SA alleviated CCO formation from the initial planting and not only in the replanting phases of ginseng cultivation. The soil repair function of CS@MSN-SA automatically changed with soil acidity. CS@MSN-SA effectively disrupted the negative feedback loop of CCO and systematically integrated soil–plant–microbe interactions. This study offered a sustainable strategy for the cultivation of ginseng and other plants.

## Data Availability

The raw data supporting the conclusions of this article will be made available by the authors, without undue reservation.

## Footnotes

1
www.bioincloud.tech
